# Investigating the Role of Rhodopsin *F45L* Mutation in Mouse Rod Photoreceptor Signaling and Survival

**DOI:** 10.1523/ENEURO.0330-22.2023

**Published:** 2023-03-02

**Authors:** Deepak Poria, Alexander V. Kolesnikov, Tae Jun Lee, David Salom, Krzysztof Palczewski, Vladimir J. Kefalov

**Affiliations:** 1 Department of Ophthalmology, Gavin Herbert Eye Institute, University of California, Irvine, CA 92697; 2 Department of Chemistry, University of California, Irvine, CA 92697; 3 Department of Physiology and Biophysics, University of California, Irvine, CA 92697; 4 Department of Molecular Biology and Biochemistry, University of California, Irvine, CA 92697; 5 Department of Ophthalmology and Visual Sciences, Washington University School of Medicine, Saint Louis, MO 63110

**Keywords:** electroretinogram, phototransduction, retinal degeneration, rhodopsin, rods

## Abstract

Rhodopsin is the critical receptor molecule which enables vertebrate rod photoreceptor cells to detect a single photon of light and initiate a cascade of molecular events leading to visual perception. Recently, it has been suggested that the F45L mutation in the transmembrane helix of rhodopsin disrupts its dimerization *in vitro*. To determine whether this mutation of rhodopsin affects its signaling properties *in vivo*, we generated knock-in mice expressing the rhodopsin F45L mutant. We then examined the function of rods in the mutant mice versus wild-type controls, using *in vivo* electroretinography and transretinal and single cell suction recordings, combined with morphologic analysis and spectrophotometry. Although we did not evaluate the effect of the F45L mutation on the state of dimerization of the rhodopsin *in vivo*, our results revealed that F45L-mutant mice exhibit normal retinal morphology, normal rod responses as measured both *in vivo* and *ex vivo*, and normal rod dark adaptation. We conclude that the F45L mutation does not affect the signaling properties of rhodopsin in its natural setting.

## Significance Statement

Absorption of a photon by the visual chromophore produces conformational changes in rhodopsin to open up a transducin-binding pocket and initiate the downstream signaling. The most abundantly expressed form of rhodopsin is its dimeric configuration, which is disrupted *in vitro* by the F45L mutation. Here, we show that mouse rods expressing mutant F45L rhodopsin exhibit no changes in sensitivity, response kinetics, or chromophore reconstitution compared with the rods of mice expressing wild-type rhodopsin. Our findings indicate that the F45L mutation does not affect the functional properties of the visual pigment rhodopsin. Future studies will be required to determine how the F45L mutation affects rhodopsin dimerization in the intact rod photoreceptors.

## Introduction

**Figure 1.**
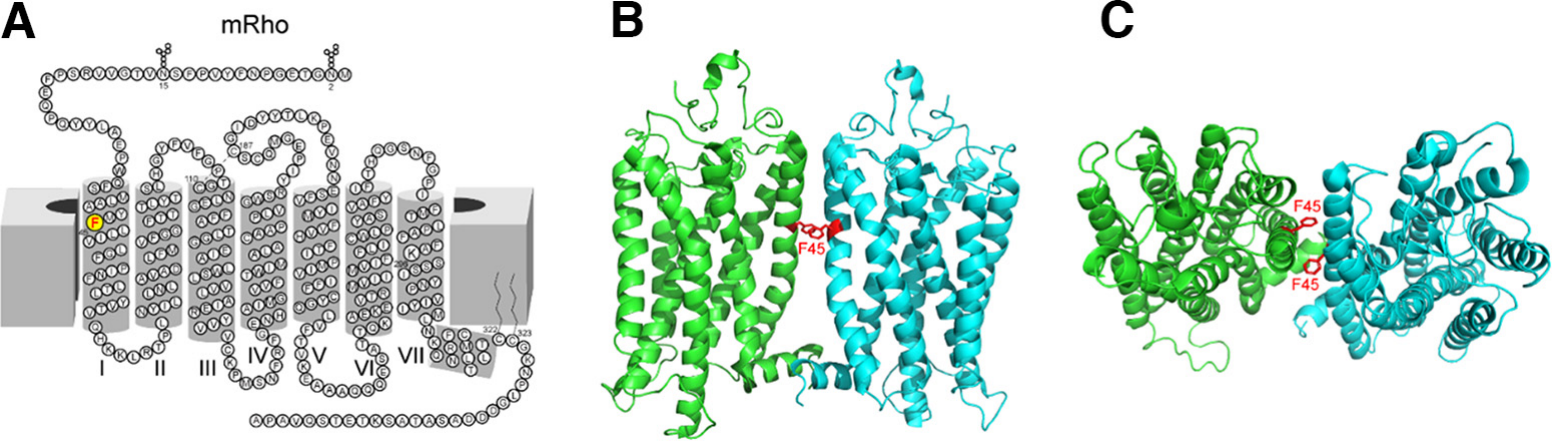
The *Rho^F45L^ knock-in* mutation. A 2D cartoon of mouse rhodopsin showing the mutation site at amino acid position 45 in the transmembrane helix I, where phenylalanine is replaced by leucine in the *Rho^F45L^ KI* mice (***A***). Side (***B***) and axial (***C***) views of the 3D structure of bovine rhodopsin’s dimer as determined by cryo-electron microscopy (PDB ID 6OFJ), highlighting the position of F45.

The visual pigment rhodopsin, a prototype G-protein-coupled receptor (GPCR; Palczewski, 2006), mediates probably the most sensitive sensory transduction, the detection of a single photon of light by the visual system (Baylor et al., 1979; Rieke, 2000). This high sensitivity is made possible by the substantial amplification of the rod transduction cascade following photoactivation of the rhodopsin chromophore (Pugh and Lamb, 1993; Arshavsky and Burns, 2014; Yue et al., 2019). It has been established that even a single rhodopsin molecule (Fig. 1*A*) expressed *in vitro* can initiate downstream intracellular signaling (Ernst et al., 2007; Whorton et al., 2007, 2008; Tsukamoto et al., 2010). These findings indicate that rhodopsin can function as a monomeric unit. However, *in vitro* purification studies have shown that rhodopsin forms oligomers, among which dimers are the most prevalent (Fig. 1*B*; Sung et al., 1991b; Fotiadis et al., 2006; Jastrzebska et al., 2006; Park et al., 2008). Moreover, when expressed abundantly, the recombinant rhodopsin exists as a dimer in cultured cells as well (Sung et al., 1991b; Kota et al., 2006). A recent study of the morphologic structure of the disk surface of the rod outer segment showed that rhodopsin molecules organize as rows of dimers on the disk membrane (Zhao et al., 2019). However, several mutations of the rhodopsin molecule, *F45L*, *V209M*, and *F220C*, have been shown to disrupt the dimerization of the protein *in vitro* (Sung et al., 1991b; Kaushal and Khorana, 1994; Ploier et al., 2016). One of these studies (Kaushal and Khorana, 1994) reported the binding affinity of monomeric rhodopsin for transducin to be compromised. These rhodopsin mutations have been detected in patients with retinal degenerative disease and previously were interpreted to be associated with the disease phenotype (Sung et al., 1991a; Berson et al., 2002). However, recent evidence ruled out a role for either the *F45L* or *F220C* mutations in retinal degeneration (Vincent et al., 2013; Lewis et al., 2020).

Studies with mutant *F45L* rhodopsin have shown that this rod visual pigment can translocate and incorporate successfully into the cell membrane and rod outer segments (Ploier et al., 2016). Specific sites in the transmembrane domains of the rhodopsin partner molecules have been speculated to interact within the dimers through various states of activation of the receptor (Fig. 1*C*; Salom et al., 2006; Scheerer et al., 2008; Cordomi and Perez, 2009; Choe et al., 2011). Additionally, the individual rhodopsin subunits distinctly rearrange within a dimer complex as compared with the single molecular state (Cordomi and Perez, 2009). These structural modifications present possibilities for alterations in target binding sites, potentially triggering allosteric mechanisms that could be involved in modulation of the rhodopsin activity.

Rhodopsin is the protypical member of GPCR subfamily A, among which negative allosteric interactions between homomeric partners have been shown previously (Springael et al., 2005; Urizar et al., 2005; Rivero-Muller et al., 2010). However, potential allosteric interactions within rhodopsin dimers and their effect on visual perception remain uncharacterized.

In this study, we sought to investigate the role of the F45 rhodopsin residue in rhodopsin signaling by studying the light response sensitivity, kinetics, and survival of rods in mice carrying the *Rho^F45L^* knock-in mutation. In the process of preparing this manuscript, another group published a study on an unrelated F45L mutant mouse line that reported some findings similar to ours (Lewis et al., 2020).

## Materials and Methods

### Animals

The *Rho^F45L^ KI* mutant mice were generated commercially (Ingenious Targeting Laboratory) on a C57Bl/6 background. The codon substitution TTC>CTC at position 45 was confirmed by sequencing. The animals were maintained in a 12 h/12 h light/dark cycle at all times. Both male and female animals of three months of age were used in the experiments, unless age is specified otherwise.

### Electrophysiology

For physiology experiments, all animals were dark-adapted overnight before the day of the experiment. For *in vivo* ERG recordings, the animals were anaesthetized using a cocktail of ketamine (100 mg/kg) and xylazine (4 mg/kg). Pupils were dilated with a drop of 1% atropine sulfate. The mouse body temperature was maintained at 37°C with a heating pad connected to a controller. Full-field ERG responses to calibrated green (530 nm) LED light were recorded from both eyes by contact corneal electrodes held in place by a drop of Gonak solution (Akorn). ERGs were recorded using a clinical ERG setup (LKC Technologies; Model UBA-4200c) adapted for mice.

Rod ERG a-wave fractional flash sensitivity (*S_f_*) was calculated from the linear part of the intensity-response curve, as follows:

$$S_{f}=\frac{R}{R_{max}\cdot I},$$

where *R* is the amplitude of the rod a-wave dim flash response, *R_max_* is the maximum amplitude of the rod a-wave response for that eye (determined at 23.5 cd·s m^−2^), and *I* is the flash strength. The sensitivity of rods was first determined in the dark. To monitor the postbleach recovery of *R_max_* and *S_f_*, more than 90% of rhodopsin was bleached with a 35-s exposure to 520-nm LED light focused at the surface of the cornea. The bleached pigment fraction was calculated with the following equation:

$$F=1-e^{\left(-I\cdot P\cdot t\right)},$$

where *F* is the fraction of rhodopsin bleached, *t* is the duration of the light exposure (s), *I* is the bleaching light intensity of 520-nm LED light (1.3 × 10^8^ photons μm^−2^ s^−1^), and *P* is the photosensitivity of mouse rods at the wavelength of peak absorbance (5.7 × 10^−9^ μm^2^; Woodruff et al., 2004). Mice were re-anesthetized once after 30 min with a lower dose of ketamine (∼1/3 of the initial dose) and a small drop of PBS solution was gently applied to their eyes with a plastic syringe to protect them from drying and to maintain contact with the recording electrodes.

For *ex vivo* transretinal recordings, the animals were euthanized with CO_2_ and then their eyes were enucleated under dim red light followed by dissection under infrared illumination. The dissection was performed in a Petri dish containing oxygenated Ames’ medium (Sigma). First, the eyeballs were cut close to the limbus, then the retina was gently detached from the posterior eye cup by tearing the sclera and retinal pigmented epithelium (RPE), using forceps. The retinas were stored in oxygenated Ames’ medium in a dark chamber at room temperature until recording. Recordings were conducted using previously described methods (Vinberg and Kefalov, 2015). The recordings were made using a closed chamber where the retina was mounted with photoreceptors facing up. The recording chamber was continuously supplied with oxygenated Ames’ medium at a flow rate of 3–5 ml/min. For isolating the photoreceptor component of the transretinal response, 50 μm DL-AP_4_ (Tocris) and 100 μm BaCl_2_ (Sigma) were included in the Ames’ medium. The chamber temperature was maintained at 35–36°C, and retinas were allowed to adapt to the chamber temperature for at least 15 min before the start of the recordings. *Ex vivo* ERG recordings were made by presenting light flashes produced by computer-controlled LEDs (Thor Labs). The signals were amplified using a differential amplifier (Warner Instruments), low-pass filtered at 300 Hz (Krohn Hite Corp.), digitized using Digidata 1440 (Molecular Devices), and recorded on a computer at a sampling frequency of 10 kHz, using pClamp 10 software.

For single-cell suction recordings from rod outer segments, following dissection of the eyes under infrared illumination, the retinas were chopped into small pieces in a dish containing oxygenated Locke’s solution (in mm: 112.5 NaCl, 3.6 KCl, 2.4 MgCl_2_, 1.2 CaCl_2_, 10 HEPES, 20 NaHCO_3_, 0.02 EDTA, 3.0 Na_2_-succinate, 0.5 Na-glutamate, 10.0 glucose, and 0.1% MEM vitamins). The retinal pieces were then transferred to an open chamber maintained at 35–36°C with a continuous supply of heated Locke’s solution at 2–3 ml/min. Borosilicate glass pipettes, pulled to ∼1-μm inner diameter over a heated filament (Sutter Instruments), fire-polished, and filled with electrode solution (in mm: 140 NaCl, 3.6 KCl, 2.4 MgCl_2_, 1.2 CaCl_2_, 3.0 HEPES, 0.02 EDTA, and 10.0 glucose; pH adjusted to 7.4 with NaOH), were used in these experiments. Single rod outer segments were approached under infrared visual control and gently drawn into the glass pipette. Recordings were made by presenting flash stimuli produced by computer-controlled LEDs (Thor Labs). Signals were amplified using Axopatch 200B, low-pass filtered at 10 Hz (Krohn Hite Corp.), digitized using Digidata 1440 (Molecular Devices), and recorded on a computer at a sampling frequency of 10 kHz, using pClamp 10 software.

### Data analysis

Data were analyzed using Clampfit 10.7 (Molecular Devices), Microsoft Excel and Origin 9.8.5 (64 bit, SR2, OriginLab) and presented as mean ± SEM *p*-values of <0.05 (Student’s *t* test) were considered significant. The intensity-response relationships data were fitted by a hyperbolic Naka–Rushton function using the following equation:

$$\frac{R}{R_{max}}=\frac{I^{n}}{I^{n}+I_{1∕2}^{n}},$$

where *R* is the flash response, *R_max_* is the maximum response amplitude, *I* is the flash intensity, *n* is the Hill coefficient, and *I_1/2_* is the intensity to produce half-saturating response. The light adaptation data were fitted by a modified Weber–Fechner function, as follows:

$$\frac{S_{f}}{S_{fDA}}=\frac{I_{0}^{n}}{I_{0}^{n}+I^{n}},$$

where *S_f_* is the rod sensitivity (as defined above), *S_f_^DA^* is the rod sensitivity in darkness, *n* is a slope factor (Hill coefficient), *I* is the background light intensity (in photons μm^−2^ s^−1^), and *I_0_* is the background light intensity needed to reduce the sensitivity to 50% of that in darkness.

### Morphology and microscopy

For morphologic studies, the eyeballs from three-, six-, and nine-month-old animals were fixed overnight in 4% paraformaldehyde at 4°C, embedded in paraffin, and then sectioned into 10-μm-thick sections. For identification of the dorsal and ventral sides of the retinas, the eyes were marked on the ventral surface of the cornea by a high-temperature cautery pen. Retinal sections were stained with hematoxylin and eosin (H&E) to label the nuclei. The stained sections were imaged at 40× magnification using an Olympus BX51 microscope. The outer and inner nuclear layer thickness was measured using ImageJ software (NIH).

### Rhodopsin measurements

Mouse eyes were enucleated in darkness under dim red light. Each eye was flash-frozen on dry ice immediately after enucleation. Rhodopsin was extracted with 20 mm HEPES, pH 7.4, containing 10 mm n-dodecyl-β-maltoside and 5 mm freshly neutralized NH_2_OH·HCl, as described previously (Palczewska et al., 2018). Briefly, the tissue was homogenized with 0.9 ml of buffer in a 2-ml Dounce tissue homogenizer (Kontes Glass Co) and shaken for 5 min at 4°C. The sample was then centrifuged at 17,200 × *g* for 5 min at 4°C. The supernatant was collected, the pellet was extracted a second time with 0.9 ml of buffer, and the combined supernatants were filtered through a 0.22-μm polyethersulfone membrane. Absorbance spectra were recorded using a Varian Cary 50-Scan UV-Vis spectrophotometer (Varian Australian Pty Ltd.); the sample was used as blank, then it was bleached for 5 min with a white-light, 875-Lumens bulb, and finally the difference absorbance spectrum was recorded immediately following a bleach. The concentration of rhodopsin was determined by the decrease in absorbance at 500 nm using the molar extinction coefficient ε_500nm_ = 42,000 M^−1^ ·cm^−1^.

## Results

### *Rho^F45L^ KI* mutation does not cause rod degeneration

**Figure 2.**
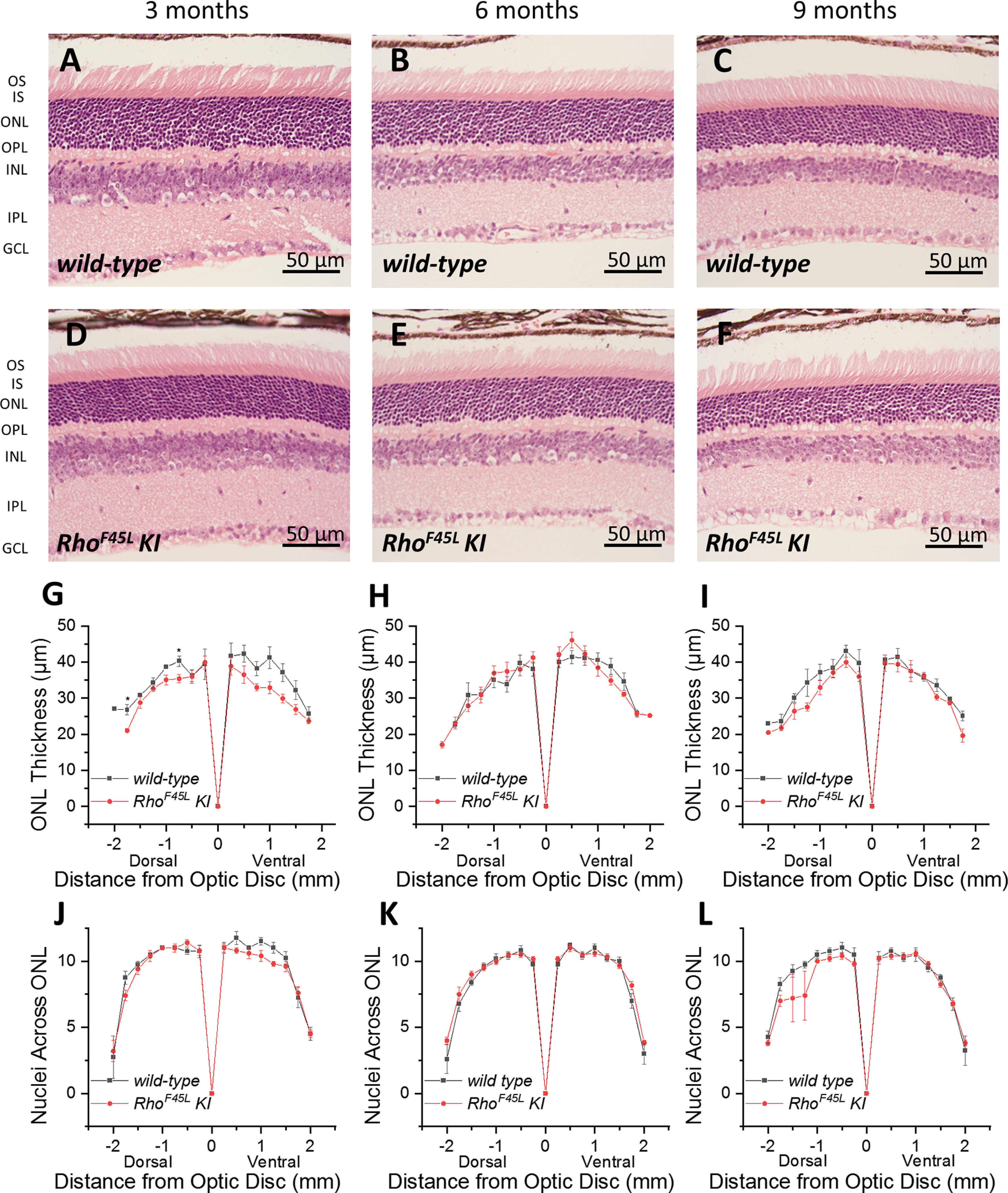
Effect of the *Rho^F45L^ knock-in* mutation on photoreceptor morphology. Representative images from retinas of three-, six-, and nine-month-old *wild-type* mice (***A*–*C***, respectively), and age-matched *Rho^F45L^ KI* mice (***D*–*F***, respectively). Quantitative spider plots of ONL thickness as a function of distance from the optic nerve disk from *wild-type* mice (black; *n* = 4 each), and from *Rho^F45L^ KI* mice (red; *n* = 5, 4, 5) measured at three-, six-, and nine-month time points (***G–I***, respectively). Quantitative spider plots of the number of photoreceptor nuclei per column in the ONL as a function of distance from the optic nerve disk from *wild-type* mice (black; *n* = 4 each), and from *Rho^F45L^ KI* mice (red; *n* = 5, 4, 5) measured at three-, six-, and nine-month time points (***J–L***, respectively). Error bars indicate SEM; *p < 0.05.

*Rho^F45L^* expressed *in vitro* has been demonstrated to retain the capability to activate transducin; however, its binding affinity to transducin was shown to be reduced (Kaushal and Khorana, 1994). Because the loss of rhodopsin leads to photoreceptor degeneration in mice (Lem et al., 1999), we speculated that a partial loss of pigment function in the *Rho^F45L^ KI* mouse line could also lead to rod death. First, we examined the retinal morphology at three different time points. We found that there were no detectable changes in the outer nuclear layer (ONL) thickness in three-, six-, and nine-month-old *wild-type* mice (Fig. 2*A–C*, respectively) or in the age-matched *Rho^F45L^ KI* mutant mice (Fig. 2*D–F*, respectively). We then quantified the ONL morphology by measuring the thickness (Fig. 2*G–I*) as well as by counting the nuclei per ONL column (Fig. 2*J–L*) at several different locations across the retina, which showed no significant differences between the two types of mice at any of the time points studied, with the exception of two different locations in the dorsal retina of three-month-old mice where we observed small but significant diminutions of 22% and 12% for the *Rho^F45L^ KI* mice (Fig. 2*G*). Thus, overall, the *Rho^F45L^ KI* mutation did not cause notable rod degeneration in the mouse retina.

### *Rho^F45L^ KI* mutation does not affect the expression of rhodopsin in rods

**Figure 3.**
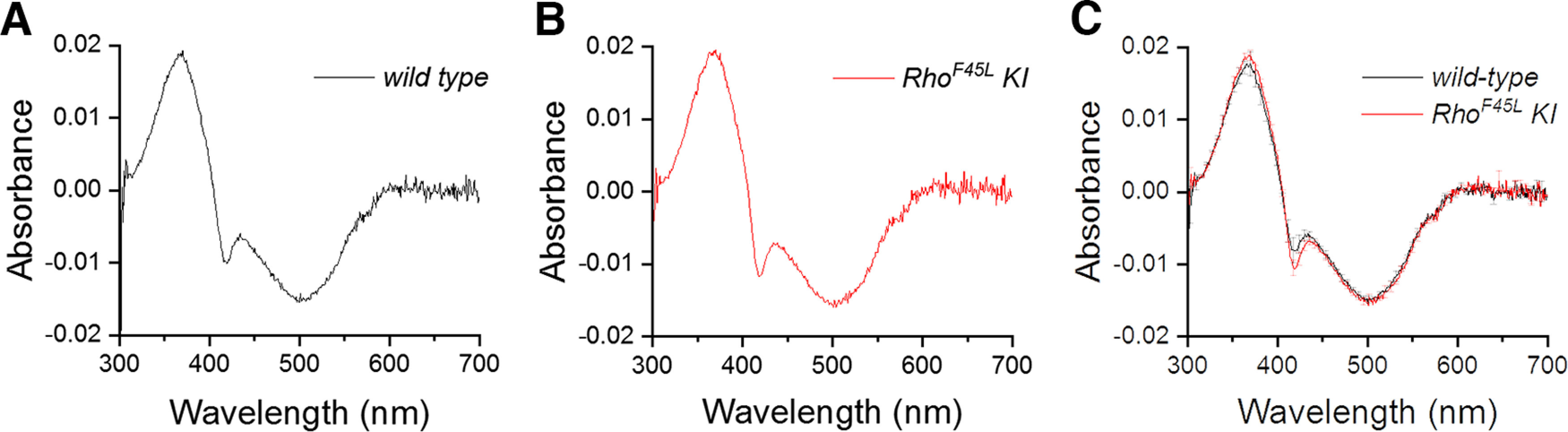
Effect of the *Rho^F45L^ knock-in* mutation on rhodopsin expression. Representative difference absorbance spectra of rhodopsin (before vs after bleaching) from *wild-type* mice (***A***) and *Rho^F45L^ KI* mice (***B***). Averaged spectra from *wild-type* mice (black; *n* = 2 eyes) and *Rho^F45L^ KI* mice (red; *n = *2 eyes; ***C***). All measurements were done from three-month-old animals.

The normal development and health of rods is strongly dependent on the proper level of expression of rhodopsin (Fulton et al., 2009; Wen et al., 2009). Our finding that the *Rho^F45L^ KI* mutant retina does not present detectable degeneration even at nine months of age suggests that the mutant rhodopsin is expressed at a normal (fully functional) level compared with that in *wild-type* rods. To evaluate rhodopsin expression directly, we measured absorbance spectra for eye extracts of rhodopsin from retinas of *wild-type* and *Rho^F45L^ KI* mutant mice. We found that the *F45L* variant exhibited peak absorbance at 500 nm, similar to *wild-type* rhodopsin (Fig. 3). There were no significant differences in the quantified rhodopsin absorbance spectra for retina samples from *Rho^F45L^ KI* mutant and *wild-type* mice, indicating that their rhodopsin levels and spectral characteristics were indistinguishable.

### *Rho^F45L^ KI* mutation does not affect the rod response

**Figure 4.**
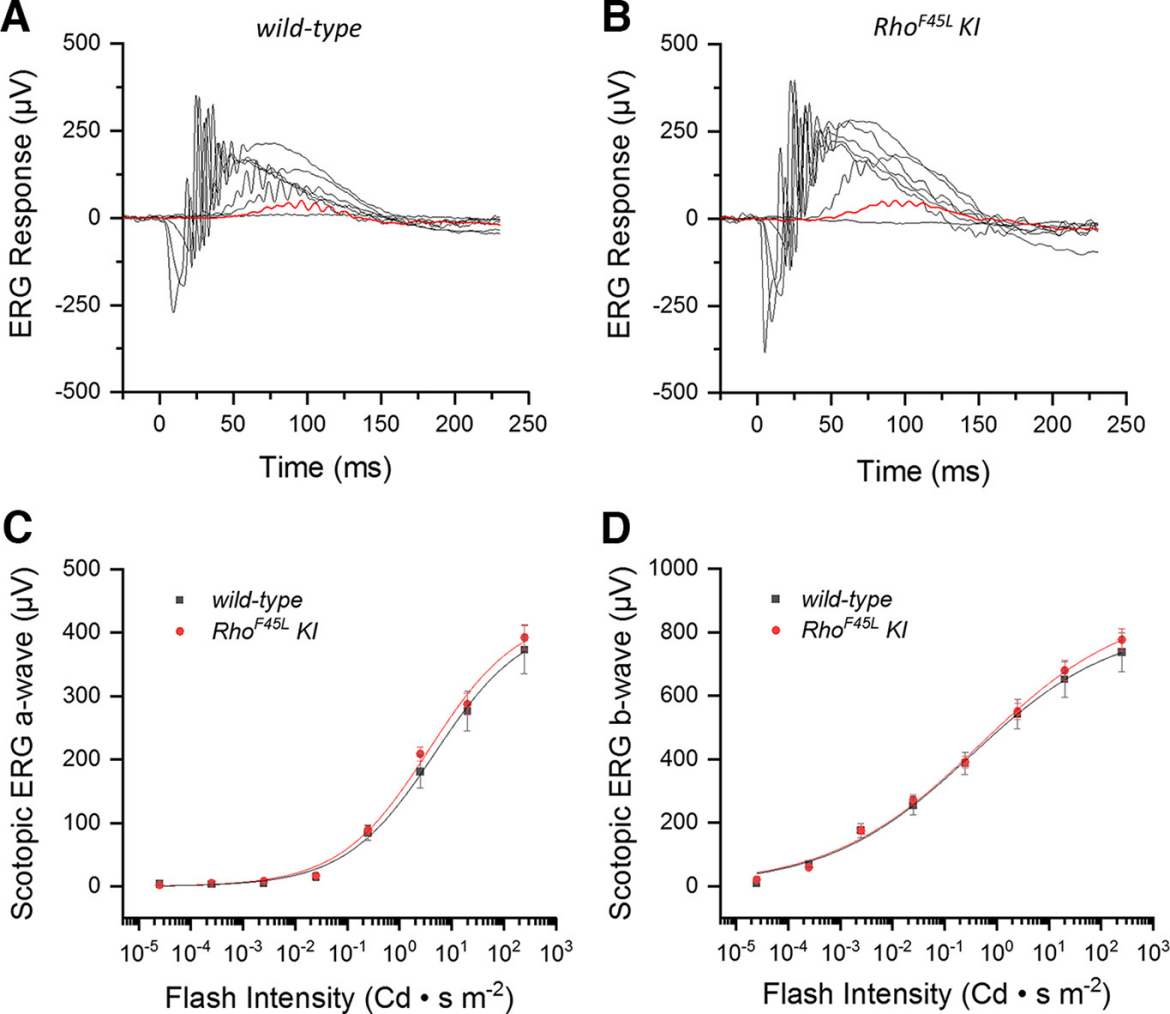
Effect of the *Rho^F45L^ knock-in* mutation on *in vivo* scotopic ERG responses. Representative families of ERG responses to flashes of increasing intensity (Cd · s m^−2^: 2.5 × 10^−5^, 2.5 × 10^−4^, 2.5 × 10^−3^, 2.5 × 10^−2^, 0.25, 2.5, 20, and 250) recorded in scotopic conditions from *wild-type* mice (***A***) and *Rho^F45L^ KI* mice (***B***). For comparison, the responses to a flash of 2.5 × 10^−4^ Cd · s m^−2^ are highlighted in red in the two panels. Population-averaged a-wave response amplitudes (***C***) and b-wave response amplitudes (***D***) from groups of *wild-type* mice (black; *n* = 5) and *Rho^F45L^ KI* mice (red; *n* = 5) are plotted together as a function of flash intensity. Error bars indicate SEM. Differences in ***C*** and ***D*** were not significant (*p* > 0.05) for all data points. All measurements were done from three-month-old mice.

We next tested whether the *Rho^F45L^ KI* mutation affected the physiological response of the rods by recording *in vivo* ERG responses under scotopic conditions. We found that the rod-driven responses of *Rho^F45L^ KI* mutant mice (Fig. 4*B*) were comparable to those of *wild-type* mice (Fig. 4*A*). Comparison of the a-wave (Fig. 4*C*) and b-wave (Fig. 4*D*) intensity-response relationships further revealed that they were essentially identical for *wild-type* and the *Rho^F45L^ KI* mutant mice. This finding was also consistent with the results of scotopic optomotor reflex experiments performed with these mice, which showed statistically indistinguishable visual acuity and contrast sensitivity for the two groups of mice (Table 1).

**Table 1:** Scotopic visual behavior test results

	Visual acuity (cyc/°)	Contrast threshold (%)	*N*
*wild type*	0.72 ± 0.04	11.9 ± 1.5	3 mice
*Rho^F45L^ KI*	0.67 ± 0.02	12.7 ± 1.2	6 mice
*p* value	>0.05	>0.05	

**Figure 5.**
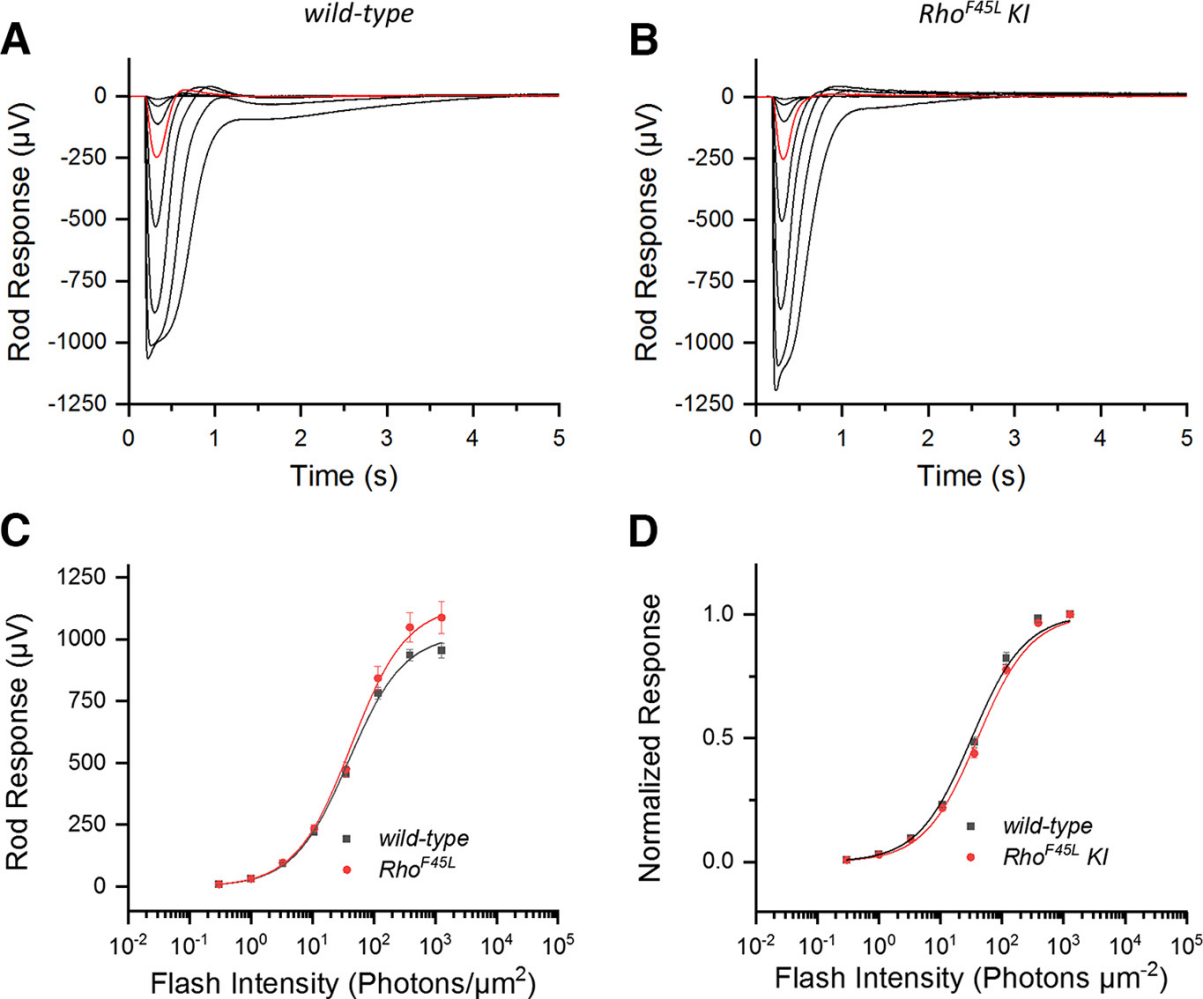
Effect of the *Rho^F45L^ knock-in* mutation on *ex vivo* ERG rod responses. Representative families of responses to flashes of increasing intensity (photons μm^−2^: 0.3, 1, 3, 10.7, 35, 117, 386, and 1271) for retinas from *wild-type* mice (***A***) and *Rho^F45L^ knock-in* mutant mice (***B***). For comparison, the responses to a flash of 35 photons μm^−2^ are highlighted in red in both panels. Average flash response amplitudes (***C***) and average normalized flash response amplitudes (***D***) for rods from *wild-type* mice (black; *n* = 5 mice, 8 retinas) and *Rho^F45L^ knock-in* mutant mice (red; *n* = 6 mice, 10 retinas) are plotted together as a function of flash intensity. Error bars indicate SEM. The lines represent curves fitted to the data using a hyperbolic Naka–Rushton function. All measurements were done from three-month-old mice.

We next turned to *ex vivo* ERG recordings that allow pharmacological manipulation of the retinal response to isolate its photoreceptor component. The rod responses recorded *ex vivo* were also similar for the *wild-type* and *Rho^F45L^ KI* mutant mice (Fig. 5*A*,*B*, respectively), and had comparable maximal amplitudes and sensitivities (Table 2). The intensity-response functions for these two groups were only marginally different (Fig. 5*C*) and the composite sets of data were evaluated as *not* statistically different (Table 2). The normalized intensity-response relationships were also indistinguishable for the *wild-type* and *Rho^F45L^ KI* mutant mice (Fig. 5*D*), demonstrating their similar photosensitivities (Table 2).

**Table 2:** Transretinal recordings analysis parameters

	R_max_ (μV)	S_f_^DA^ (× 10^−3^ phot^−1^ μm^2^)	I_1/2_ (phot μm^−2^)	*N*
*wild type*	954 ± 30	22 ± 1	39 ± 4	5 mice, 8 retinas
*Rho^F45L^ KI*	1088 ± 64	21 ± 1	46 ± 4	6 mice, 10 retinas
*p* value	0.08	0.40	0.16	

R_max_, saturated response amplitude measured at the plateau.

S_f_^DA^, dark-adapted sensitivity.

I_1/2_, intensity required to produce half of the saturated response.

**Figure 6.**
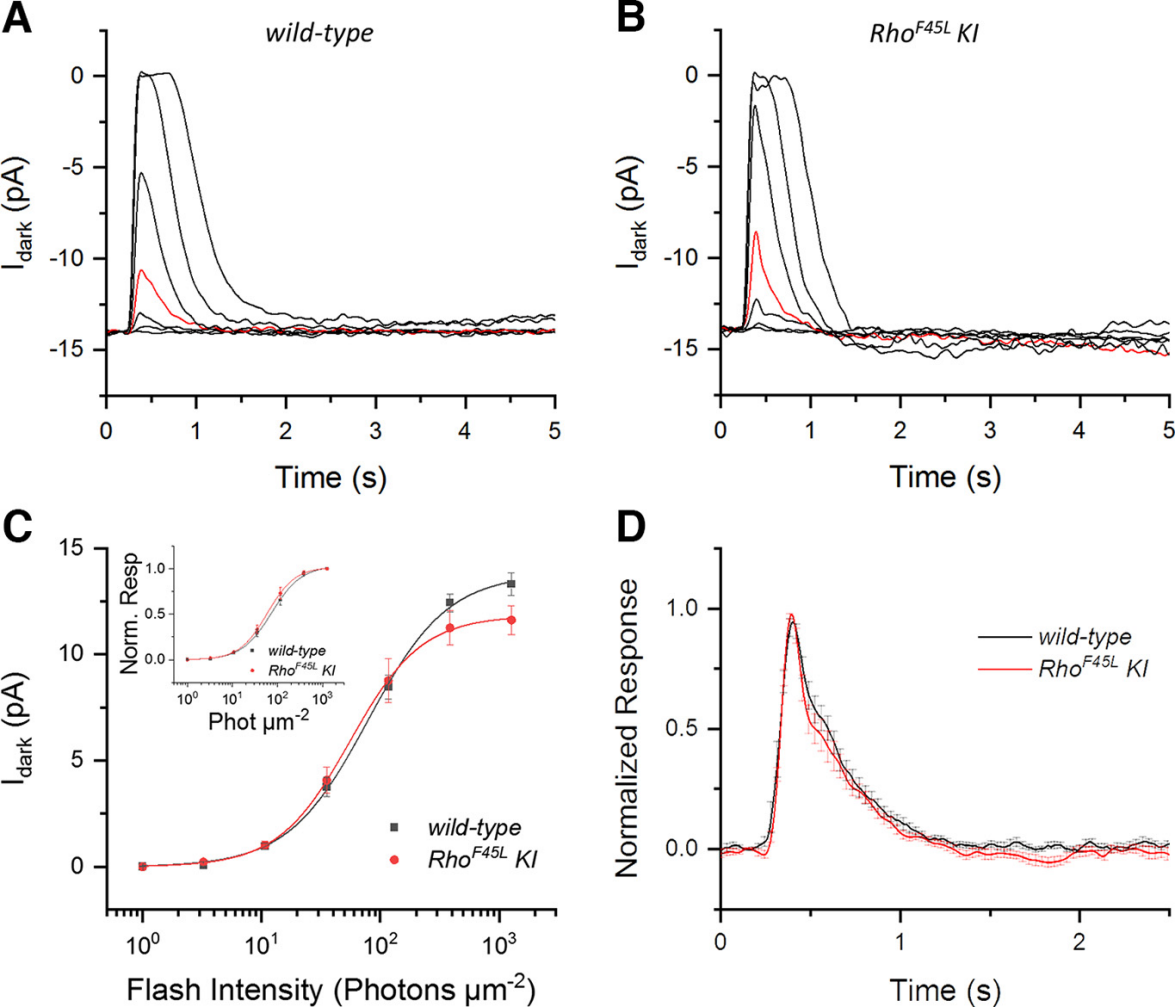
Effect of the *Rho^F45L^ knock-in* mutation on individual rod responses. Representative families of responses of individual rods to flashes of increasing intensity (photons μm^−2^: 1, 3, 10.7, 35, 117, 386, and 1271); (***A***) rods from *wild-type* mice, and (***B***) rods from *Rho^F45L^ knock-in* mutant mice. For comparison, the responses to a flash of 35 photons μm^−2^ are highlighted in red in the two panels. Population-averaged flash response amplitudes (***C***) and averaged normalized flash response amplitudes (***C***, inset) for rods from *wild-type* mice (black; *n* = 14 cells), and rods from *Rho^F45L^ knock-in* mutant mice (red; *n* = 13 cells) plotted together as a function of flash intensity. Error bars indicate SEM. The lines represent curves fitted to the data points using a Naka–Rushton function. ***D***, Normalized averaged dim flash responses for rods from *wild-type* mice (black; *n* = 10 cells), and from *Rho^F45L^ knock-in* mutant mice (red; *n* = 13 cells) plotted together for comparison of response kinetics. All measurements were done from three-month-old mice.

To study the kinetics of rod responses, we compared dim flash responses from single rod outer segments of *wild-type* and *Rho^F45L^ KI* mutant mice. As expected, the response amplitudes and sensitivities were comparable between the two groups (Fig. 6*A–C*; Table 3). We also found that the rod dim*-*flash response kinetics were indistinguishable between *wild-type* and *Rho^F45L^ KI* mutant mice (Fig. 6*D*), with similar times to peak, integration times, and recovery time constants for the two groups (Table 3). Thus, the overall data indicate that the *Rho^F45L^ KI* mutation did not affect the rod light response.

**Table 3:** Rod outer segment suction recordings analysis parameters

	I_dark_ (pA)	S_f_^DA^ (× 10^−3^ phot^−1^ μm^2^)	I_1/2_ (phot μm^−2^)	T_p_ (ms)	T_int_ (ms)	τ_rec_ (ms)	*N*
*wild type*	13 ± 1	8 ± 1	89 ± 14	215 ± 12	941 ± 60	273 ± 24	14 cells
*Rho^F45L^ KI*	12 ± 1	10 ± 1	76 ± 15	227 ± 10	965 ± 39	357 ± 61	13 cells
*p* value	0.06	0.52	0.55	0.43	0.74	0.22	

I_dark_, saturated response amplitude measured at the plateau.

S_f_^DA^, dark-adapted sensitivity.

I_1/2_, intensity required to produce half of the saturated response.

T_p_, time to peak of a dim flash response.

T_int_, integration time of the response.

τ_rec_, recovery time constant during response shut off.

### *Rho^F45L^ KI* mutation does not affect dark adaptation of rods

**Figure 7.**
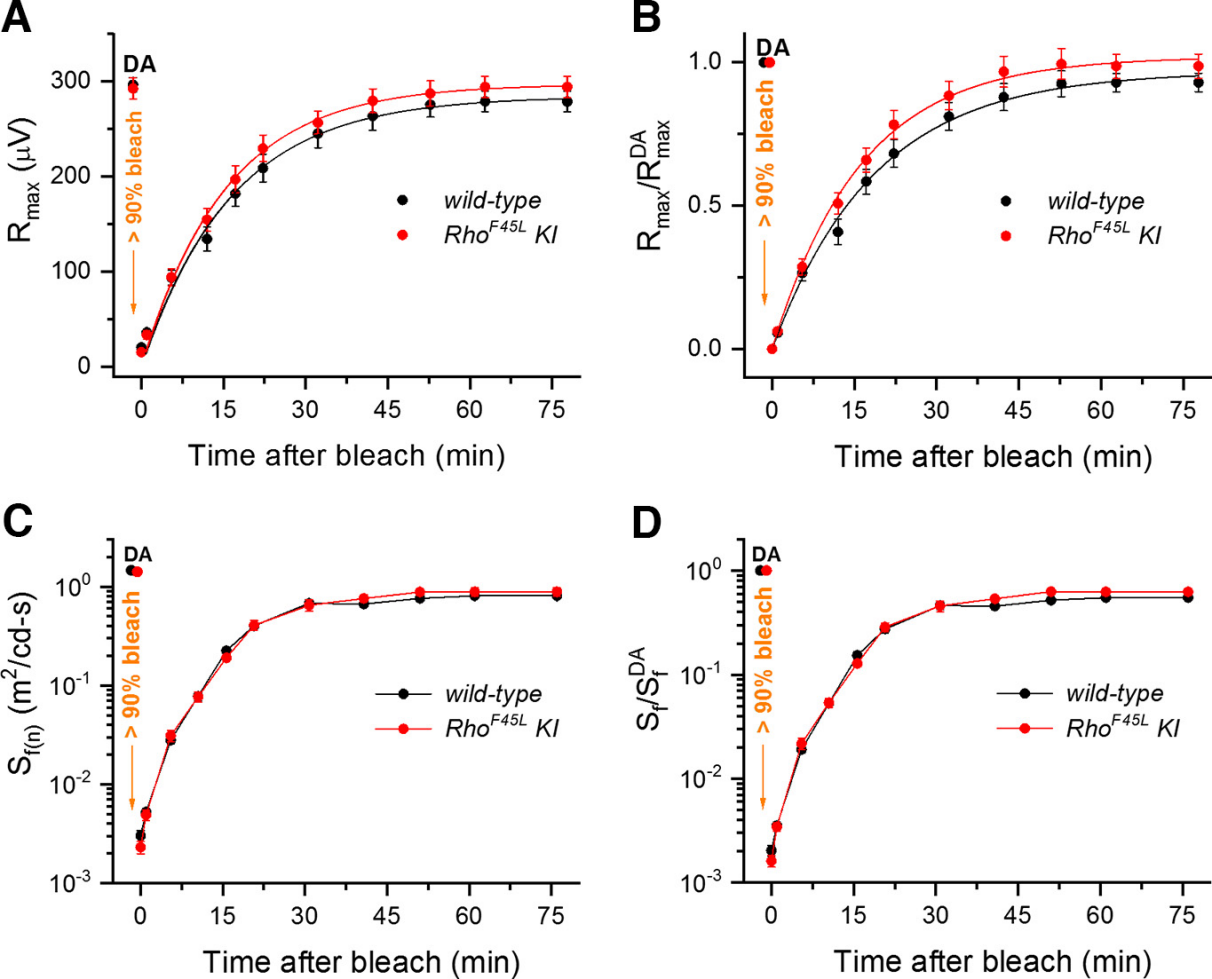
Effect of the *Rho^F45L^ knock-in* mutation on dark adaptation of rods. Recovery of absolute (***A***) and normalized (***B***) scotopic ERG maximal a-wave amplitudes (R_max_; mean ± SEM) after bleaching >90% of rhodopsin in eyes of *wild-type* mice (black, *n* = 12) and *Rho^F45L^ knock-in* mutant mice (red, *n* = 12). Bleaching was achieved by a 35-s illumination with bright 520 nm LED light at time 0. R_max_^DA^ refers to the prebleach maximal response in the dark (DA). Averaged data points were fitted with single exponential functions yielding time constants of 18.7 ± 0.9 and 16.2 ± 0.6 min for *wild-type* and *Rho^F45L^ knock-in* mice, respectively. Recovery of absolute (***C***) and normalized (***D***) scotopic ERG a-wave flash sensitivity (*S*_f_; mean ± SEM) after bleaching >90% of rhodopsin in the same *wild-type* mice (black, *n* = 12) or *Rho^F45L^ knock-in* mice (red, *n* = 12). *S*_f_^DA^ designates the sensitivity of dark-adapted (DA) rods. All measurements were done from three-month-old mice.

Finally, to investigate a possible impact of the *Rho^F45L^* mutation on the ability of rods to process their rhodopsin photointermediates and regenerate their visual pigment after its substantial bleaching, we measured the kinetics of rod dark adaptation *in vivo* (Fig. 7*A–D*). Under these conditions, the rate of rod dark adaptation is determined by the speed of recycling of the spent visual chromophore (all-*trans*-retinal) back to its initial 11-*cis*-retinal form in the canonical RPE-dependent retinoid (visual) cycle. In accordance with the unchanged intensity-response relationship (Fig. 4*C*), the maximal dark-adapted (DA) scotopic ERG a-wave amplitude, R_max_, was not affected by the *Rho^F45L^* substitution in this separate group of two-month-old mice (297 ± 8 μV for controls vs 293 ± 11 μV for mutants, *n* = 12 in each case, *p *>* *0.05; Fig. 7*A*). Rod ERG a-wave photosensitivity, S_f_, was also identical in the two groups (1.47 ± 0.04 m^2^ cd·s^−1^ for controls vs 1.40 ± 0.04 m^2^ cd·s^−1^ for mutants, *n* = 12 in each case, *p *>* *0.05; Fig. 7*C*). The single-exponential recovery of rod-driven ERG a-wave response following exposure of the eyes to brief bright light (estimated to bleach > 90% of rhodopsin) was also unaltered, with its final postbleach levels reaching 81% and 89% for *wild-type* and mutant mice, respectively (Fig. 7*B*); and the lack of difference was confirmed for the comparison of the recovery of normalized scotopic ERG a-wave sensitivity for *wild-type* and *Rho^F45L^* animals (Fig. 7*D*). We conclude that the *Rho^F45L^* mutation does not affect the kinetics of regeneration of rhodopsin and the dark adaptation of rods, consistent with the normal pigment levels and photoresponses in *Rho^F45L^* mice in the dark.

## Discussion

In this study, we investigated the possible effect of a Phe to Leu substitution mutation *F45L* in rhodopsin on the morphology and visual function of rod photoreceptors in the mouse retina. Our results demonstrate that the *Rho^F45L^* mutation does not lead to any changes in the function of rhodopsin; thus, the physiology, health, and survival of the rods remain unchanged. These results are consistent with recent studies by Lewis et al., 2020; where they showed that mice expressing rhodopsin *F45L-mutants* or *F220C-mutants* exhibited no change in rod physiology, protein distribution, morphology, or survival.

Our finding that the quantified absorbance of the *F45L-mutant* rhodopsin is unchanged from the wild-type rhodopsin indicates that the absolute level of rhodopsin remains unaltered in the *Rho^F45L^ KI* rods. Additionally, we found that the light response amplitude and sensitivity of rods from *Rho^F45L^ KI* mutant mice were essentially the same as those from wild*-*type mice, suggesting that the activation phase of the response is unchanged in the *Rho^F45L^* mutants. The amplification of the rod phototransduction cascade is directly proportional to the level of G-protein transducin α-subunit in the outer segments of mammalian rods (Arshavsky et al., 2002; Sokolov et al., 2002) and depends on the overall binding affinity of transducin heterotrimer to rhodopsin (Kolesnikov et al., 2011). Thus, together these findings suggest that, not only do these mutant rods express normal levels of rhodopsin and transducin leading to efficient amplification of the transduction cascade, but also that the rhodopsin-transducin interaction and binding affinity remain normal.

We also found that the time course of dark adaptation of the rods from the mutant *Rho^F45L^ KI* mice after near complete bleaching of their visual pigment was indistinguishable from that of the rods from wild-type mice. This novel observation indicates that the overall kinetics of pigment regeneration *in vivo* are not affected by the *F45L* mutation of rhodopsin. Dark adaptation of rod photoreceptors is a complex process that involves the release and reduction of the spent all-*trans*-retinal from photoactivated rhodopsin, followed by its recycling to 11-*cis*-retinal in the retinal pigmented epithelium (RPE), its return to photoreceptors, and finally binding to free opsin and formation of a Schiff base to reconstitute the ground-state rhodopsin molecule (Lamb and Pugh, 2004). The overall speed of this process is limited by the supply of fresh chromophore from the RPE to the rods (Lamb and Pugh, 2004; Wang et al., 2014). However, modulation of the thermal decay of photoactivated rhodopsin intermediates by G-protein-coupled receptor kinase 1 and arrestin 1 (Frederiksen et al., 2016) and its phosphorylation status (Kolesnikov et al., 2017) can also affect the overall time course of dark adaptation of rods. Thus, our observation that rod dark adaptation in *Rho^F45L^ KI* mice is unchanged indicates that the chromophore release, its re-isomerization in the RPE, and subsequent binding of freshly formed 11-*cis*-retinal to mutant opsin all remain normal in these animals.

Overall, our findings rule out the possibility that the *Rho^F45L^* mutation exerts a functionally significant allosteric modulation of rhodopsin either during signaling or during pigment regeneration in mouse rods. Additionally, we show that the mutant rods remain healthy for up to several months of age, suggesting that the *Rho^F45L^* mutation does not give rise to any pathologic conditions leading to photoreceptor death. These findings are consistent with recent studies and, in conjunction, support the emerging view that the *F45L* point mutation is not implicated in such hereditary diseases as retinitis pigmentosa (Vincent et al., 2013; Lewis et al., 2020). The *F45L* mutation detected in a few retinitis pigmentosa patients diagnosed earlier could possibly be a consequence of this mutation occurring coincidentally with other unidentified mutations leading to the disease, as several newer mutations implicated in inherited retinal degenerations have been identified in the following decades (Sung et al., 1991a; Berson et al., 2002). Future studies will have to evaluate the effect of the F45L mutation on rhodopsin dimerization *in vivo* or use alternative or complementary methods of disrupting rhodopsin dimers (Jastrzebska et al., 2006; Getter et al., 2021) to further investigate the possible role of allosteric interactions between rhodopsin monomers in disk membranes in photoreceptor signaling.

## Synthesis

Reviewing Editor: Matthew Grubb, King’s College London Decisions are customarily a result of the Reviewing Editor and the peer reviewers coming together and discussing their recommendations until a consensus is reached. When revisions are invited, a fact-based synthesis statement explaining their decision and outlining what is needed to prepare a revision will be listed below. The following reviewer(s) agreed to reveal their identity: NONE.

Synthesis:

Please address all of the Reviewers’ points below, being especially careful about statements and conclusions regarding the status of rhodopsin dimerisation in the F45L mouse. Also please note in relation to Reviewer 2’s comment #2 that additional analysis involving nuclei counting would be ideal here, but is not absolutely necessary if the limitations of measuring ONL thickness are explicitly detailed in the manuscript. For Reviewer 2’s comment #3, please consider swapping images for examples where the RPE is present (if such example images are available; this is not absolutely necessary and certainly does not require new sample preparation), and provide separate annotation for OS and IS layers.

Reviewer 1:

This manuscript addresses the mouse phenotype of the F45L rhodopsin mutation that has been previously loosely associated with retinitis pigmentosa and in vitro defects in rhodopsin dimerization. The authors find essentially no consequences of the F45L mutation in a knockin mouse model, which replicates the findings of a previously published study on an independently generated F45L mutant mouse (Lewis, et al. 2020). The experimental methodology of the current study is clean and thorough. However, there is an issue with the authors’ conclusions. In several places (noted below), the authors assume that the F45L mutation affects rhodopsin dimerization in vivo. However, this has only been shown in in vitro assays (Ploier, et al. 2016) in which the concentration of rhodopsin would be several orders of magnitude less abundant than in the outer segment. To justify the authors’ conclusions as they currently stand, an in vivo assessment of the dimerization status of F45L rhodopsin would have to be performed (such as with atomic force microscopy or cryo-EM). Unless such an experiment is performed, the authors should soften their conclusions and not to base them on an axiomatic assumption that dimerization was affected in their mouse. This includes the following instances:

1) The abstract reads as if the authors come to a negative conclusion regarding a physiological role of rhodopsin dimerization. It should be clarified that it is unknown whether rhodopsin dimerization is actually disrupted in their F45L mouse model.

2) Significance Statement: the entire statement builds to the conclusion that “Our findings rule out the possibility that the dimerization affects the functional properties of the visual pigment rhodopsin”.

3) Line 45: “However, several mutations of the rhodopsin molecule, F45L, V209M and F220C, have been shown to disrupt the dimerization of the protein”. It should be clarified that this dimerization disruption has only been shown in vitro.

4) Line 53: “Studies with mutant F45L rhodopsin have shown that this monomeric form of the rod visual pigment can translocate and incorporate successfully into ... rod outer segments”. The dimerization status of F45L rhodopsin in these in vivo experiments was not known.

5) Line 66: “In this study, we sought to investigate the physiological role of rhodopsin dimerization by studying the light response sensitivity, kinetics, and survival of rods in mice carrying the RhoF45L knock‐in mutation, which is known to disrupt the dimerization of rhodopsin (Ploier et al., 2016); and to test the hypothesis that rhodopsin dimerization plays a role in allosteric modulation of the G protein‐signaling”. Without assaying the dimerization status of F45L rhodopsin in vivo, these hypotheses were never tested.

6) Line 226: “Our results demonstrate that the RhoF45L mutation, which is expected to disrupt the oligomerization of this GPCR receptor and produce monomeric visual pigment molecules, does not lead to any changes in the function of rhodopsin...”.

This reviewer would like to stress that these issues should not preclude these data from publication, but they must be fixed in revision before publication is possible.

Reviewer 2

The manuscript fits the category used “negative results”. The findings in this work replicate a previously published paper. I don’t find that as a negative since the reproducibility of science is a concern. The extensive characterization in this work with the previous published conclusively show that the F45L mutation of rhodopsin in murine models does not affect photoreceptor cells.

Specific Comments:

1. The animal model used is not described. What strain background was used? Details on model generation should be included.

2. Photoreceptor degeneration was assessed using H&E, and the ONL length was quantified to confirm the degeneration. The length of the ONL may differ in tissues due to fixation, which could be the reason for the observed difference in the dorsal retina area (Line 169-170; figure 2G). I would advise the author to consider counting the outer nuclei instead.

3. Minor - RPE layer missing in H and E, Fig. 2F

4. For the in vivo and ex vivo recordings, the data is convincing except that the age is not clear. It is important to mention at what age these recordings are done. If the recordings are done at 3 months, would it be possible to have the recordings at 9 months?

5. There is an emphasis on dimerization and F45L mutation. In the abstract, ...”it has been suggested that the F45L mutation of rhodopsin disrupts its dimerization”. In the significance statement, the authors are emphatic in stating “The dimeric configuration is the most abundantly expressed form of rhodopsin which is disrupted by the F45L mutation” Which one is it? “suggested” or the second statement?

6. In the significance statement “here, we show that the F45L mutant rods expressing monomeric rhodopsin...” Also in the discussion “Overall, our findings rule out the possibility that the F45L mutation exerts an allosteric modulation of rhodopsin within its dimeric complex...” The authors did not address the status of rhodopsin in this manuscript even though the rationale for the work appears to have started from this. In any case, the authors do not show or prove the monomeric status or allosteric modulation of rhodopsin. These statements should be removed.

## Author Response

Dear Dr. Grubb:

We are delighted that our manuscript entitled “Investigating the role of rhodopsin F45L mutation in mouse rod photoreceptor signaling and survival” was deemed potentially suitable for publication in eNeuro, pending revisions. We have revised the manuscript to address the suggestions of Reviewer 1, and a point-by-point reply is provided below. We believe that the revised manuscript is substantially improved and hope that it is now suitable for publication in eNeuro.

Reviewer 1:

As outlined in my previous review, the experimental data presented in this manuscript are of high quality and worthy of publication. However, as also outlined in my initial review, the authors maintain an essentially misleading premise that their in vivo study relates to rhodopsin dimerization. While I understand that this may have been the initial intention of this work, they do not provide any evidence whether or not dimerization is actually affected in the F45L mutant mouse. It is essential to state somewhere that the status of rhodopsin dimerization in this mouse is unknown and to tone down the implications that their study relates to rhodopsin dimerization. Some related points that need to be fixed are detailed below:

1) The first half of the abstract still focuses on rhodopsin dimerization and poses the premise: “it remains unclear whether dimerization plays any physiological role in modulating the function of the visual pigment, rhodopsin”. By framing their study this way, they misguide the reader into believing that their study will address this question. This would be warranted if the authors provided data regarding the status of rhodopsin dimerization in the F45L mouse, which they do not. Thus, the abstract has to be rewritten to reflect what is actually shown in this study.

Response: We had made an effort to address the Reviewer’s concern about implications about the status of rhodopsin dimerization from our study. We feel that a complete removal of any reference to the suggested role of F45L mutation in rhodopsin dimerization would obscure the initial motivation for our study. Instead, in the current revision we have opted to explicitly state that this issue is not experimentally addressed in our manuscript, while also addressing the specific comments of Reviewer 1. We hope that this will address adequately the Reviewer’s legitimate concern about inferring any role of F45L in rhodopsin dimerization from our study.

2) The significance statement still suffers from the same issue described above for the abstract where half of the text focuses on rhodopsin dimerization, which is not addressed.

Response: We revised the Significance Statement to deemphasize rhodopsin dimerization and also to state that the dimerization status of F45L rhodopsin has not been determined as part of our study.

3) The authors end the introduction with the line “In this study, we sought to investigate the role of the F45 rhodopsin residue in rhodopsin signaling by studying the light response sensitivity, kinetics, and survival of rods in mice carrying the RhoF45L knock-in mutation, which is known to disrupt the dimerization of 2 rhodopsin in vitro”. Again, framing the Introduction in this way calls for the authors to directly address the status of rhodopsin dimerization in the F45L mouse.

Response: We have removed the reference to rhodopsin dimerization from the final sentence of the

Introduction.

4) The authors start the discussion with the line “Our results demonstrate that the RhoF45L mutation, which in vitro disrupts the oligomerization of this GPCR receptor and produces monomeric visual pigment molecules, does not lead to any changes in the function of rhodopsin”. This, again, misleads the reader into thinking that their study addressed the role of rhodopsin dimerization.

Response: We have removed the reference to rhodopsin dimerization from that sentence.

5) Also in the discussion, “Overall, our findings rule out the possibility that the RhoF45L mutation exerts an allosteric modulation of rhodopsin within its dimer complex...”. This line suggests that the F45L mutation does not affect the dimer complex.

Response: We have removed the reference to rhodopsin dimerization from that sentence.

6) The last line of the discussion, “Future studies will have to make use of alternative or complementary methods of disrupting rhodopsin dimers to further investigate the possible role of allosteric interactions between rhodopsin monomers in disc membranes in photoreceptor signaling.” This line suggests that the authors know whether or not their approach of disrupting rhodopsin dimers was successful and that they did indeed investigate the role of dimerization in their current study.

Response: Our intention was to state that future studies will need to be done to evaluate the role of rhodopsin dimerization in rod phototransduction. We have revised the sentence in question in an effort to clarify this point.

Reviewer 2:

The authors have addressed my comments constructively.

Response: Thank you.

Thank you very much for your consideration

## References

[B1] Arshavsky VY, Burns ME (2014) Current understanding of signal amplification in phototransduction. Cell Logist 4:e29390. 10.4161/cl.29390 25279249 PMC4160332

[B2] Arshavsky VY, Lamb TD, Pugh EN Jr (2002) G proteins and phototransduction. Annu Rev Physiol 64:153–187. 10.1146/annurev.physiol.64.082701.102229 11826267

[B3] Baylor DA, Lamb TD, Yau KW (1979) Responses of retinal rods to single photons. J Physiol 288:613–634. 112243 PMC1281447

[B4] Berson EL, Rosner B, Weigel-DiFranco C, Dryja TP, Sandberg MA (2002) Disease progression in patients with dominant retinitis pigmentosa and rhodopsin mutations. Invest Ophthalmol Vis Sci 43:3027–3036. 12202526

[B5] Choe HW, Kim YJ, Park JH, Morizumi T, Pai EF, Krauss N, Hofmann KP, Scheerer P, Ernst OP (2011) Crystal structure of metarhodopsin II. Nature 471:651–655. 10.1038/nature09789 21389988

[B6] Cordomi A, Perez JJ (2009) Structural rearrangements of rhodopsin subunits in a dimer complex: a molecular dynamics simulation study. J Biomol Struct Dyn 27:127–147. 19583439 10.1080/07391102.2009.10507303

[B7] Ernst OP, Gramse V, Kolbe M, Hofmann KP, Heck M (2007) Monomeric G protein-coupled receptor rhodopsin in solution activates its G protein transducin at the diffusion limit. Proc Natl Acad Sci U S A 104:10859–10864. 17578920 10.1073/pnas.0701967104 PMC1904172

[B8] Fotiadis D, Jastrzebska B, Philippsen A, Muller DJ, Palczewski K, Engel A (2006) Structure of the rhodopsin dimer: a working model for G-protein-coupled receptors. Curr Opin Struct Biol 16:252–259. 16567090 10.1016/j.sbi.2006.03.013

[B9] Frederiksen R, Nymark S, Kolesnikov AV, Berry JD, Adler L, Koutalos Y, Kefalov VJ, Cornwall MC (2016) Rhodopsin kinase and arrestin binding control the decay of photoactivated rhodopsin and dark adaptation of mouse rods. J Gen Physiol 148:1–11. 27353443 10.1085/jgp.201511538 PMC4924931

[B10] Fulton AB, Hansen RM, Moskowitz A (2009) Development of rod function in term born and former preterm subjects. Optom Vis Sci 86:E653–E658. 19483509 10.1097/OPX.0b013e3181a6a237 PMC2822655

[B11] Getter T, Kemp Vinberg F, Palczewski K (2021) Identification of small-molecule allosteric modulators that act as enhancers/disrupters of rhodopsin oligomerization. J Biol Chem 297:101401. 34774799 10.1016/j.jbc.2021.101401 PMC8665362

[B12] Jastrzebska B, Fotiadis D, Jang GF, Stenkamp RE, Engel A, Palczewski K (2006) Functional and structural characterization of rhodopsin oligomers. J Biol Chem 281:11917–11922. 16495215 10.1074/jbc.M600422200 PMC1618955

[B13] Kaushal S, Khorana HG (1994) Structure and function in rhodopsin. 7. Point mutations associated with autosomal dominant retinitis pigmentosa. Biochemistry 33:6121–6128. 8193125 10.1021/bi00186a011

[B14] Kolesnikov AV, Rikimaru L, Hennig AK, Lukasiewicz PD, Fliesler SJ, Govardovskii VI, Kefalov VJ, Kisselev OG (2011) G-protein betagamma-complex is crucial for efficient signal amplification in vision. J Neurosci 31:8067–8077. 21632928 10.1523/JNEUROSCI.0174-11.2011 PMC3118088

[B15] Kolesnikov AV, Orban T, Jin H, Brooks C, Hofmann L, Dong Z, Sokolov M, Palczewski K, Kefalov VJ (2017) Dephosphorylation by protein phosphatase 2A regulates visual pigment regeneration and the dark adaptation of mammalian photoreceptors. Proc Natl Acad Sci U S A 114:E9675–E9684. 29078372 10.1073/pnas.1712405114 PMC5692576

[B16] Kota P, Reeves PJ, Rajbhandary UL, Khorana HG (2006) Opsin is present as dimers in COS1 cells: identification of amino acids at the dimeric interface. Proc Natl Acad Sci U S A 103:3054–3059. 16492774 10.1073/pnas.0510982103 PMC1413904

[B17] Lamb TD, Pugh EN Jr (2004) Dark adaptation and the retinoid cycle of vision. Prog Retin Eye Res 23:307–380. 15177205 10.1016/j.preteyeres.2004.03.001

[B18] Lem J, Krasnoperova NV, Calvert PD, Kosaras B, Cameron DA, Nicolo M, Makino CL, Sidman RL (1999) Morphological, physiological, and biochemical changes in rhodopsin knockout mice. Proc Natl Acad Sci U S A 96:736–741. 9892703 10.1073/pnas.96.2.736 PMC15206

[B19] Lewis TR, Shores CR, Cady MA, Hao Y, Arshavsky VY, Burns ME (2020) The F220C and F45L rhodopsin mutations identified in retinitis pigmentosa patients do not cause pathology in mice. Sci Rep 10:7538. 10.1038/s41598-020-64437-y 32371886 PMC7200662

[B20] Palczewska G, Stremplewski P, Suh S, Alexander N, Salom D, Dong Z, Ruminski D, Choi EH, Sears AE, Kern TS, Wojtkowski M, Palczewski K (2018) Two-photon imaging of the mammalian retina with ultrafast pulsing laser. JCI Insight 3:e121555. 30185665 10.1172/jci.insight.121555 PMC6171813

[B21] Palczewski K (2006) G protein-coupled receptor rhodopsin. Annu Rev Biochem 75:743–767. 10.1146/annurev.biochem.75.103004.142743 16756510 PMC1560097

[B22] Park JH, Scheerer P, Hofmann KP, Choe HW, Ernst OP (2008) Crystal structure of the ligand-free G-protein-coupled receptor opsin. Nature 454:183–187. 10.1038/nature07063 18563085

[B23] Ploier B, Caro LN, Morizumi T, Pandey K, Pearring JN, Goren MA, Finnemann SC, Graumann J, Arshavsky VY, Dittman JS, Ernst OP, Menon AK (2016) Dimerization deficiency of enigmatic retinitis pigmentosa-linked rhodopsin mutants. Nat Commun 7:12832. 10.1038/ncomms12832 27694816 PMC5059438

[B24] Pugh EN Jr, Lamb TD (1993) Amplification and kinetics of the activation steps in phototransduction. Biochim Biophys Acta 1141:111–149. 8382952 10.1016/0005-2728(93)90038-h

[B25] Rieke F (2000) Mechanisms of single-photon detection in rod photoreceptors. Methods Enzymol 316:186–202. 10.1016/s0076-6879(00)16724-2 10800676

[B26] Rivero-Muller A, Chou YY, Ji I, Lajic S, Hanyaloglu AC, Jonas K, Rahman N, Ji TH, Huhtaniemi I (2010) Rescue of defective G protein-coupled receptor function in vivo by intermolecular cooperation. Proc Natl Acad Sci U S A 107:2319–2324. 20080658 10.1073/pnas.0906695106 PMC2836644

[B27] Salom D, Lodowski DT, Stenkamp RE, Le Trong I, Golczak M, Jastrzebska B, Harris T, Ballesteros JA, Palczewski K (2006) Crystal structure of a photoactivated deprotonated intermediate of rhodopsin. Proc Natl Acad Sci U S A 103:16123–16128. 17060607 10.1073/pnas.0608022103 PMC1637547

[B28] Scheerer P, Park JH, Hildebrand PW, Kim YJ, Krauss N, Choe HW, Hofmann KP, Ernst OP (2008) Crystal structure of opsin in its G-protein-interacting conformation. Nature 455:497–502. 10.1038/nature07330 18818650

[B29] Sokolov M, Lyubarsky AL, Strissel KJ, Savchenko AB, Govardovskii VI, Pugh EN Jr, Arshavsky VY (2002) Massive light-driven translocation of transducin between the two major compartments of rod cells: a novel mechanism of light adaptation. Neuron 34:95–106. 11931744 10.1016/s0896-6273(02)00636-0

[B30] Springael JY, Urizar E, Parmentier M (2005) Dimerization of chemokine receptors and its functional consequences. Cytokine Growth Factor Rev 16:611–623. 15979374 10.1016/j.cytogfr.2005.05.005

[B31] Sung CH, Davenport CM, Hennessey JC, Maumenee IH, Jacobson SG, Heckenlively JR, Nowakowski R, Fishman G, Gouras P, Nathans J (1991a) Rhodopsin mutations in autosomal dominant retinitis pigmentosa. Proc Natl Acad Sci U S A 88:6481–6485. 10.1073/pnas.88.15.6481 1862076 PMC52109

[B32] Sung CH, Schneider BG, Agarwal N, Papermaster DS, Nathans J (1991b) Functional heterogeneity of mutant rhodopsins responsible for autosomal dominant retinitis pigmentosa. Proc Natl Acad Sci U S A 88:8840–8844. 1924344 10.1073/pnas.88.19.8840 PMC52606

[B33] Tsukamoto H, Sinha A, DeWitt M, Farrens DL (2010) Monomeric rhodopsin is the minimal functional unit required for arrestin binding. J Mol Biol 399:501–511. 20417217 10.1016/j.jmb.2010.04.029 PMC3848883

[B34] Urizar E, Montanelli L, Loy T, Bonomi M, Swillens S, Gales C, Bouvier M, Smits G, Vassart G, Costagliola S (2005) Glycoprotein hormone receptors: link between receptor homodimerization and negative cooperativity. EMBO J 24:1954–1964. 15889138 10.1038/sj.emboj.7600686 PMC1142614

[B35] Vinberg F, Kefalov V (2015) Simultaneous ex vivo functional testing of two retinas by in vivo electroretinogram system. J Vis Exp (99):e52855. 10.3791/52855 PMC4469378 25992809

[B36] Vincent AL, Carroll J, Fishman GA, Sauer A, Sharp D, Summerfelt P, Williams V, Dubis AM, Kohl S, Wong F (2013) Rhodopsin F45L allele does not cause autosomal dominant retinitis pigmentosa in a large Caucasian family. Transl Vis Sci Technol 2:4. 10.1167/tvst.2.2.4 PMC3763889 24049715

[B37] Wang JS, Nymark S, Frederiksen R, Estevez ME, Shen SQ, Corbo JC, Cornwall MC, Kefalov VJ (2014) Chromophore supply rate-limits mammalian photoreceptor dark adaptation. J Neurosci 34:11212–11221. 25143602 10.1523/JNEUROSCI.1245-14.2014 PMC4138333

[B38] Wen XH, Shen L, Brush RS, Michaud N, Al-Ubaidi MR, Gurevich VV, Hamm HE, Lem J, Dibenedetto E, Anderson RE, Makino CL (2009) Overexpression of rhodopsin alters the structure and photoresponse of rod photoreceptors. Biophys J 96:939–950. 19186132 10.1016/j.bpj.2008.10.016 PMC2716671

[B39] Whorton MR, Bokoch MP, Rasmussen SG, Huang B, Zare RN, Kobilka B, Sunahara RK (2007) A monomeric G protein-coupled receptor isolated in a high-density lipoprotein particle efficiently activates its G protein. Proc Natl Acad Sci U S A 104:7682–7687. 17452637 10.1073/pnas.0611448104 PMC1863461

[B40] Whorton MR, Jastrzebska B, Park PS, Fotiadis D, Engel A, Palczewski K, Sunahara RK (2008) Efficient coupling of transducin to monomeric rhodopsin in a phospholipid bilayer. J Biol Chem 283:4387–4394. 18033822 10.1074/jbc.M703346200 PMC2651572

[B41] Woodruff ML, Lem J, Fain GL (2004) Early receptor current of wild-type and transducin knockout mice: photosensitivity and light-induced Ca2+ release. J Physiol 557:821–828. 15073279 10.1113/jphysiol.2004.064014 PMC1665159

[B42] Yue WWS, Silverman D, Ren X, Frederiksen R, Sakai K, Yamashita T, Shichida Y, Cornwall MC, Chen J, Yau KW (2019) Elementary response triggered by transducin in retinal rods. Proc Natl Acad Sci U S A 116:5144–5153. 30796193 10.1073/pnas.1817781116 PMC6421417

[B43] Zhao DY, Poge M, Morizumi T, Gulati S, Van Eps N, Zhang J, Miszta P, Filipek S, Mahamid J, Plitzko JM, Baumeister W, Ernst OP, Palczewski K (2019) Cryo-EM structure of the native rhodopsin dimer in nanodiscs. J Biol Chem 294:14215–14230. 31399513 10.1074/jbc.RA119.010089 PMC6768649

